# Pre-amplification as a method for improvement of quantitative RT-PCR analysis of circulating miRNAs

**DOI:** 10.11613/BM.2021.010901

**Published:** 2020-12-15

**Authors:** Ankica Sekovanić, Adrijana Dorotić, Jasna Jurasović, Daria Pašalić, Jelena Kovačić, Sandra Stasenko, Tatjana Mioč, Martina Piasek, Tatjana Orct

**Affiliations:** 1 Institute for Medical Research and Occupational Health, Zagreb, Croatia; 2 Department of Medical Laboratory Diagnostics, Sveti Duh University Hospital, Zagreb, Croatia; 3 Department of Medical Chemistry, Biochemistry and Clinical Chemistry, University of Zagreb, School of Medicine, Zagreb, Croatia; 4 Department of Gynecology and Obstetrics, Merkur University Hospital, Zagreb, Croatia

**Keywords:** blood plasma, circulating microRNAs, epigenetics, pre-amplification, RT-PCR

## Abstract

**Introduction:**

The assessment of circulating miRNAs is challenging and still limited due to their low concentrations, small size and lack of reference values in human biological samples. Pre-amplification of complementary DNAs may facilitate reliable miRNA quantification. The aim of our study was to evaluate the efficacy of pre-amplification as a step to increase the sensitivity of qPCR analysis for five candidate circulating miRNAs presumably related to toxic metals and cigarette smoke exposure: miR-1537, miR-190b, miR-16, miR-21, and miR-146a.

**Materials and methods:**

Candidate miRNAs expression was analysed in plasma samples of 19 mother-newborn pairs. For isolation, transcription, pre-amplification and qPCR quantification kits and protocols by Qiagen (Hilden, Germany) were used. Paired t-test or Wilcoxon rank test were used to compare miRNAs expression levels with and without a pre-amplification step prior to qPCR, separately in maternal and cord plasma. Intraclass correlation (ICC) was calculated as an agreement measure between procedures for each miRNA.

**Results:**

Pre-amplification facilitated the detection of all assayed miRNAs with an overall cycle threshold (C_T_) improvement of 6.6 ± 0.89 (P < 0.05). Excellent ICCs (> 0.90) were found between data for preamplified and not preamplified miR-16, miR-21 and miR-146a. However, these correlations for low expressed miR-190b were moderate (0.79 in maternal; 0.61 in cord plasma) and poor for miR-1537 (0.49 in maternal; no correlation in cord plasma).

**Conclusion:**

Pre-amplification is a useful, necessary step in the analysis of miR-1537 and miR-190b as a reliable procedure facilitating extracellular miRNA expression detection in human plasma by real-time PCR quantification.

## Introduction

Micro RNAs (miRNAs) are short single-stranded, non-coding endogenous RNA molecules that contain 18 to 22 nucleotides. They regulate the expression of specific messenger RNA (mRNA), mostly by binding to the complementary mRNA sequence, resulting in translational repression or direct mRNA degradation and gene silencing. miRNAs play essential roles in a wide range of biological processes, including cell proliferation, differentiation, migration, and apoptosis (*1*). Circulating miRNAs have the potential to serve as epigenetic markers in most common chronic diseases as well as environmental exposure to toxic metals and cigarette smoke (*2*, *3*). The development of circulating miRNA-based diagnostics therefore requires reliable methods in determining circulating miRNAs in peripheral blood plasma or serum with adequate precision and sensitivity. Many pre-analytical and analytical parameters remain incompletely defined and may affect the quantification of circulating miRNAs as well as the interpretation of data. These parameters refer to the overall analysis process, from the first step that includes the biological sample type (serum *vs*. plasma) to the collection and isolation of miRNA protocols, and methods for miRNA detection, normalization, and possible application of the pre-amplification procedures (*4*-*8*). Pre-amplification is a method that allows for the analysis of genes with a limited amount of nucleic acids by increasing the number of copies of nucleotide sequences in the reaction prior to polymerase chain reaction (PCR) analysis while maintaining target amplification specificity (*8*-*10*). This study evaluates the efficiency of pre-amplification before the quantitative PCR (qPCR) analysis of five miRNAs: miR-1537, miR-190b, miR-16, miR-21, and miR-146a in maternal and cord plasma. Our hypothesis was that pre-amplification increases the sensitivity of the analysis of the candidate miRNAs related to environmental exposure to toxic metals and cigarette smoke.

## Materials and methods

### Sample collection

This methodological study was conducted on biological samples collected from a subgroup of 19 healthy mother-newborn pairs recruited during 2018 at the maternity ward of Merkur University Hospital in Zagreb, Croatia. It is part of a wider epidemiological cross-sectional cohort study within a national research project (HRZZ-IP-2016-06-1998) reviewed by the appointed ethics committees of the participating institutions: Institute for Medical Research and Occupational Health, Merkur University Hospital, and University of Zagreb School of Medicine. All study participants signed informed consent forms and the research protocols on the collection and use of biological samples complied with the principles of the Helsinki Declaration.

Maternal and cord blood samples were collected after birth in vacutainer tubes (Becton-Dickinson, NJ, USA) with K_2_-EDTA anticoagulant. Within 2 hours, samples were centrifuged (3000xrpm, 20 min) and the resulting plasma was transferred into DNase/RNase-free microtubes (Sarstedt, Nümbrecht, Germany) and stored at - 80 °C until RNAs isolation.

### miRNA isolation and reverse transcription to cDNA

Micro RNAs were isolated from 200 µL plasma using the miRNeasy Serum/Plasma Kit (Qiagen, Hilden, Germany) and protocol. After phenol/guanidine-based lysis, 5.6 x 10^8^ copies of synthetic cel-miR-39 (miRNeasy Serum/Plasma Spike-in Control) was added and the lysate separated by addition of chloroform. The concentration and quality of isolated mRNA enriched with miRNAs were checked on a NanoPhotometer P360 (Implen, München, Germany). Reverse transcription was performed on a ThermoMixer C (Eppendorf, Hamburg, Germany) by Qiagen miScript II RT Kit in 10 mL reaction volume with 5 mL of miRNAs during 1 hour at 37 °C and 5 min at 95 °C, and stored at -20 °C until analysis.

### Pre-amplification of cDNA

For the target-specific pre-amplification, miScript primers (miR-16, miR-21, miR-146a, miR-190b, miR-1537) and miScript PreAMP PCR Kit (Qiagen, Hilden, Germany) were used in a 12.5 mL reaction volume with 2.5 mL of diluted cDNA (1:4). Pre-amplification conditions were: initial activation step 15 min at 95 °C and 2-step cycling (denaturation 30 s at 94 °C, annealing/extension 3 min at 60 °C) in a total of 12 cycles using a GeneAmp PCR System 2700 (Applied Biosystems, Waltham, USA).

### Real-time PCR quantification (qPCR)

Dilution for pre-amplification cDNA was 1:19, and for not preamplified cDNA 1:10. Real-time PCR quantification was performed on an AB7500 (Applied Biosystems, Waltham, USA) using a Custom miScript miRNA PCR array with 96 wells and miScript SYBR Green PCR Kit (Qiagen, Hilden, Germany) with 8.5 mL of diluted cDNA in the master mix. The qPCR conditions were: initial activation 15 min at 95 °C and 3-step cycling (denaturation 15 s at 94 °C, annealing 30 s at 55 °C and extension 30 s at 70 °C) in a total of 40 cycles. After real-time qPCR, amplification signals were computed with 7500 software v2.0.6 (Applied Biosystems), and cycle threshold (C_T_) determined. We normalized the data across samples using a cel-miR-39, *i.e.* ∆C_T_ was calculated as C_T_-miRNA – C_T_-cel-miR-39.

### Statistical analysis

Statistical analysis was performed using statistical software R, version 3.5.0 (R Foundation for Statistical Computing, Vienna, Austria). The normality of variables was tested by the Kolmogorov–Smirnov test, and significance was set at 5%. Data were presented as median (min-max) or mean ± SD. C_T_ values ≤ 35.4 were considered as successful expressions. Differences between not preamplified and preamplified samples were tested by paired t-test or Wilcoxon signed rank test. ∆C_T_ values between PreAMP *vs.* No PreAMP (direct qPCR) were compared using Spearman’s coefficient ρ, and as a measure of absolute agreement, intraclass correlation coefficient (ICC) with 95% confidence interval (CI) was calculated based on single-rating two-way random effects model (equivalent to two-way mixed-effects model in this case) for each miRNA. For ICC calculation, all C_T_ values > 35.4 were set to 36, *i.e.* as the critical number of cycles + 1, and agreement was evaluated as poor (< 0.5), moderate (0.5-0.75), good (0.75-0.9), or excellent (> 0.9) (*6*).

## Results

Our results of direct qPCR analysis showed that the ratio of successful expressions for miR-1537 and miR-190b was 5/19 and 12/19 for maternal plasma, and 2/19 and 13/19 for cord plasma, while miR-16, miR-21 and miR-146a were expressed in all of the analysed samples. The expression of miR-1537 in PreAMP samples had a success ratio of 18/19 and 17/19 in maternal and cord plasma, while miR-190b was expressed in all of the samples (Table 1). Pre-amplification facilitated the detection of all assayed candidate miRNAs with an overall mean C_T_ improvement of 6.6 ± 0.89 (P < 0.050) *vs*. No PreAMP C_T_ values.

**Table 1:** qPCR analysis results for candidate miRNAs in not preamplified (no PreAMP) and preamplified (PreAMP) plasma samples

**Maternal plasma**					
**Circulating miRNAs**	**No PreAMP**		**PreAMP**		**C_T_ improvement**
	**N**	**C_T_**	**N**	**C_T_**	
miR-1537	5	34.7 (32.6-34.9)	18	28.8 (27.2-34.6)*	6.3 (4.4-6.6)
miR-190b	12	34.6 (32.7-35.4)	19	28.8 (26.6-32.4)*	6.4 (2.1-8.2)
miR-16	19	23.6 (21.2-32.0)	19	16.4 (13.7-25.1)*	7.0 (5.3-8.6)
miR-21	19	24.1 (21.0-27.3)	19	17.6 (14.5-20.6)*	6.5 (4.9-7.8)
miR-146a	19	26.8 (24.3-32.3)	19	19.5 (17.6-25.8)*	6.7 (5.3-7.6)
**Umbilical cord plasma**					
**Circulating miRNAs**	**No PreAMP**		**PreAMP**		**C_T_ improvement**
	**N**	**C_T_**	**N**	**C_T_**	
miR-1537^†^	2	35.3 (35.2-35.3)	17	28.7 (26.0-35.4)	7.2 (6.6-7.7)
miR-190b	13	34.0 (33.4-35.3)	19	28.2 (26.6-33.6)*	6.1 (3.9-8.3)
miR-16^‡^	19	23.4 (20.9-30.8)	19	16.6 (14.6-24.4)*	6.7 (4.9-7.5)
miR-21	19	24.5 (23.2-28.9)	19	18.0 (16.0-23.7)*	6.6 (5.2-7.2)
miR-146a	19	26.7 (24.9-32.5)	19	20.1 (18.5-26.6)*	6.7 (5.9-7.4)
Values are presented as median (min-max). C_T_ - cycle threshold. PreAMP - pre-amplification. C_T_ improvement was calculated as difference between individual C_T_ values of No PreAMP and PreAMP samples. *Statistically significant difference (at P < 0.05) between No PreAMP and PreAMP samples obtained by paired t-test. ^‡^Difference between No PreAMP and PreAMP samples tested by Wilcoxon signed rank test. ^†^Difference between No PreAMP and PreAMP samples were not tested due to a small number of expressed No PreAMP samples.					

High correlations were found for miR-16, miR-21 and miR-146a (P < 0.001) between the analysed methods. For miR-190b, the correlation was 0.84 and 0.72 in maternal and cord plasma (P < 0.001), while no significant correlation (P = 0.440) was found for miR-1537 (Table 2).

**Table 2:** Spearman’s correlation coefficients (ρ_s_) for candidate miRNAs ∆C_T_ values between not preamplified (No PreAMP) and preamplified (PreAMP) plasma samples

**Maternal plasma**				
**Circulating miRNAs**	**No PreAMP** **∆C_T_**	**PreAMP** **∆C_T_**	**ρ_s_**	**P**
miR-1537	11.2±2.50	12.2±2.20	0.20	0.423
miR-190b	10.7±2.52	11.3±1.87	0.84	< 0.001
miR-16	0.76±1.09	0.47±1.09	0.96	< 0.001
miR-21	- 0.16±1.59	0.17±1.64	0.94	< 0.001
miR-146a	2.9±1.10	2.9±1.10	0.97	< 0.001
**Umbilical cord plasma**				
**Circulating miRNAs**	**No PreAMP** **∆C_T_**	**PreAMP** **∆C_T_**	**ρ_s_**	**P**
miR-1537	11.4±2.36	12.3±2.13	-0.19	0.444
miR-190b	10.3±2.13	10.8 ±1.29	0.72	0.001
miR-16	-0.17±0.98	-0.22±0.98	0.87	< 0.001
miR-21	0.64±1.00	0.81±1.02	0.92	< 0.001
miR-146a	3.1±0.60	3.1±0.64	0.85	< 0.001
Data are presented as mean ± standard deviation. N = 19 mother-newborn pairs. C_T_ - cycle threshold. ∆C_T_ - normalized C_T_ values. PreAMP - pre-amplification. ρ_s_ - Spearman’s correlation coefficients. C_T_ values reported > 35.4 were set as 36. ∆C_T_ were calculated as follows: C_T_-miRNA – C_T_-cel-miR-39. P < 0.05 was considered statistically significant.				

**Figure 1:**
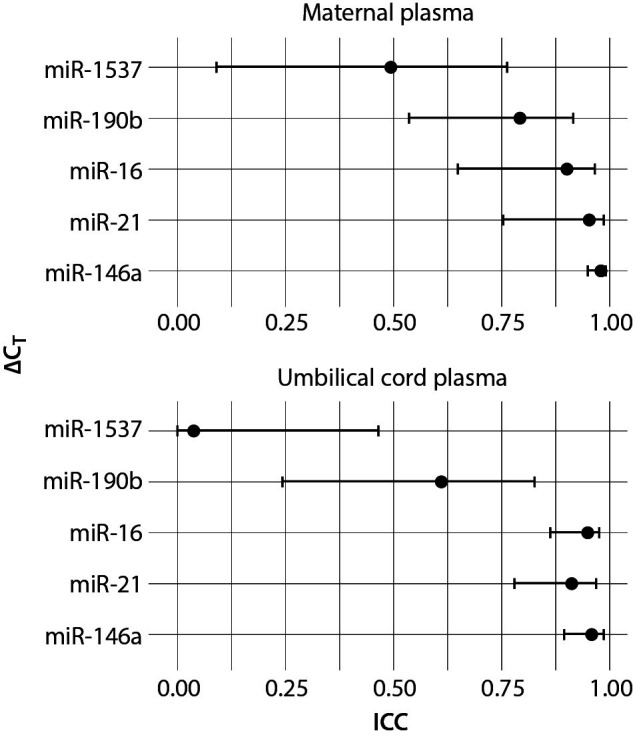
Intraclass correlation coefficient (ICC) with 95% confidence interval between qPCR miRNA expression levels in not preamplified and premplified maternal and umbilical cord plasma samples. ICC closer to 1 indicates better agreement. Negative confidence interval limits were trimmed at 0. ∆C_T_ - normalized C_T_ values.

The ICC showed excellent agreement (> 0.90) for miR-16, miR-21, and miR-146a in maternal and cord plasma. Agreement for miR-190b was moderate (0.79 in maternal and 0.61 in cord plasma), while the agreement for miR-1537 was poor (0.49 in maternal and 0.03 in cord plasma) (Figure 1).

## Discussion

The most common problem in miRNA quantification is an insufficient number of nucleotide sequence copies in the reaction that can be solved by using a pre-amplification step before qPCR analysis to increase the miRNA yield (*6*, *8*-*10*). This study showed that the pre-amplification applied before qPCR analysis improved the sensitivity, *i.e.* C_T_ values of five candidate miRNAs, without compromising the analytical specificity. Our results show that the yields of miR-1537 and miR-190b in plasma are too low for direct qPCR analysis without a pre-amplification step, whereas miR-16, miR-21, and miR-146a could be determined both without and with pre-amplification obtaining maximal yield and high agreement.

To the best of our knowledge, no recommendations state that circulating miRNAs in plasma micronomes require pre-amplification and this depends on the researcher/s assessment of the obtained results and available funds. One of the messages that we want to send out to the scientific community through this paper is that the pre-amplification technique for extracellular miRNAs should be standardized and not left merely to the investigator’s choice. It would be practical to establish databases with information on the recommended pre-amplification steps in various biological samples. Our work is guided precisely by such an idea. We, therefore, consider our study a small but important contribution to decision-making regarding the use of pre-amplification during the analysis of the micronome in human plasma, either of maternal of foetal origin as we found no differences between C_T_ values of both specimen types. This study also provides an original contribution to risk assessments of environmental exposure or any other factor/s on miRNAs expression as epigenetic markers in vulnerable population groups of mother-newborn pairs. The major weakness of this study is its small sample size and a limited number of studied candidate miRNAs. Additionally, the use of a single imputation of missing values (> 35.4 or undetermined) may lead to biased estimates in case of data under the limit of detection or high missing data rates (*6*), as was the case with miR-1537 expression. These results should therefore be interpreted with caution and future studies should explore solutions or possible bias in case of undetermined miRNA expression.

To conclude, we recommend the use of a pre-amplification process as a necessary step in miR-1537 and miR-190b analysis, whereas it is not required in miR-146a, miR-16, and/or miR-21 assessment. More evidence-based data are necessary in this regard, with the possible systematisation of such results to be used as guidelines in future studies with the identification of circulating plasma miRNAs for various purposes to see whether it is necessary or not to apply pre-amplification before qPCR analysis.
